# A simple scoring model for advanced colorectal neoplasm in asymptomatic subjects aged 40–49 years

**DOI:** 10.1186/s12876-016-0562-9

**Published:** 2017-01-09

**Authors:** Yoo Mi Park, Hee Sun Kim, Jae Jun Park, Su Jung Baik, Young Hoon Youn, Jie-Hyun Kim, Hyojin Park

**Affiliations:** 1 Department of Internal Medicine, Gangnam Severance Hospital, Yonsei University College of Medicine, 211 Eonjuro, Gangnam-gu, Seoul, 135-720 South Korea; 2 Health Promotion Center, Gangnam Severance Hospital, Yonsei University College of Medicine, Seoul, South Korea

**Keywords:** Adenoma, Colonoscopy, Colorectal neoplasm, Risk assessment, Screening

## Abstract

**Background:**

Limited data are available for advanced colorectal neoplasm in asymptomatic individuals aged 40–49 years. We aimed to identify risk factors and develop a simple prediction model for advanced colorectal neoplasm in these persons.

**Methods:**

Clinical data were collected on 2781 asymptomatic subjects aged 40–49 years who underwent colonoscopy for routine health examination. Subjects were randomly allocated to a development or validation set. Logistic regression analysis was used to determine predictors of advanced colorectal neoplasm.

**Results:**

The prevalence of overall and advanced colorectal neoplasm was 20.2 and 2.5% respectively. Older age (45–49 years), male sex, positive serology of *Helicobacter pylori*, and high triglyceride and low high-density lipoprotein (HDL) levels were independently associated with an increased risk of advanced colorectal neoplasm. BMI (body mass index) was not significant in multivariable analysis. We developed a simple scoring model for advanced colorectal neoplasm (range 0–9). A cutoff of ≥4 defined 43% of subjects as high risk for advanced colorectal neoplasm (sensitivity, 79%; specificity, 58%; area under the receiver operating curve = 0.72) in the validation datasets.

**Conclusion:**

Older age (45–49 years), male sex, positive serology of *H. pylori*, high triglyceride level, and low HDL level were identified as independent risk factors for advanced colorectal neoplasm.

## Background

Colorectal cancer (CRC) is one of the most prevalent cancers with a high incidence in Western countries [1]. The incidence of CRC has increased approximately 2–4 times in the past decades in many Asian countries [2, 3]. Because most CRC develops through the adenoma–carcinoma sequence [4], CRC can be prevented by colonoscopy with polypectomy of premalignant lesions. With respect to the screening age of CRC, since there is a significant increase in the incidence of CRC during the sixth decade of life, most CRC guidelines recommend that screening colonoscopy begin at age 50 for asymptomatic, average-risk individuals [5–7].

Although the frequency of colon cancer over 50 years has decreased in Western countries, the incidence of CRC has increased in 12 per 100,000 in 1987 to 18 per 100,000 in 2006, which is a 50% increase over 20 years among persons aged 40–44 years [8]. Persons younger than 50 years of age account for 7–9% of those diagnosed with CRC, and they tend to present with more advanced disease and have a less favorable prognosis than those older than 50 [9]. These clinical characteristics of younger persons with CRC may be associated with a time delay in diagnosis; hence, current CRC screening guidelines exclude the younger population. To minimize the amount of younger CRC patients who are not captured by the screening program, a tailored approach based on the risk of advanced adenoma that considers cost-effectiveness may be needed because the prevalence of colonic neoplasm in this age group is significantly lower than that in those aged ≥50 years [10, 11].

Regarding advanced adenoma, which is the most reliable target lesion for CRC screening, several markers, including the westernization of lifestyles, obesity, and metabolic syndrome, have been reported as risk factors for this neoplasm in the younger population, especially in those aged 40–49 years. Although these risk factors can be tailored in CRC screening for the younger age group, the systematic application of scattered risk factors may be limited in daily clinical practice. Therefore, the aim of this study was to identify risk factors and develop a simple prediction model for advanced colorectal neoplasm in asymptomatic individuals aged 40–49 years.

## Methods

This study was conducted in two steps. First, we identified risk factors associated with the occurrence of advanced colorectal neoplasm in the development set, and developed a simple scoring model for the prediction of advanced colorectal neoplasm based on independent risk factors. Subsequently, we evaluated the clinical effectiveness of a prediction model for advanced neoplasm in the validation set. This study was approved by the institutional review board of Gangnam Severance Hospital.

### Study population

A cross-sectional study was conducted with 2781 asymptomatic adults aged 40–49 years who underwent screening colonoscopy for the first time from January 2008 to January 2012 at the Health Promotion Center of the Gangnam Severance Hospital in Seoul, South Korea. We excluded the following criteria: 1) previous colorectal examinations, including colonoscopy, sigmoidoscopy, or barium enema; 2) colonoscopies that had inadequate bowel preparation and did not reach the cecum; 3) individuals who had a personal history of colorectal neoplasm and inflammatory bowel disease; 4) a history of colorectal surgery, and 5) any symptoms, weight loss, anemia, or bleeding. Subjects were randomly allocated to a development or validation set in a 2:1 ratio.

### Colonoscopy

Bowel preparation was performed using 4 L of polyethylene glycol solution, and subjects underwent a 3-day dietary restriction. The quality of bowel preparation was determined by the physician who used the following descriptors: excellent, good, fair/adequate, inadequate, and poor. Those that were inadequate and poor were labeled as poor preparation and were not included in the final analysis. Colonoscopies were performed by four endoscopists who had performed a minimum of 1000 colonoscopies, and all endoscopists were gastroenterology fellowship-trained and board-certified in their respective field. All examinations were performed using a standard video colonoscope (CF-H260AI; Olympus, Tokyo, Japan). All detected polyps were biopsied or removed. All polyp characteristics such as the size, number, shape, and location were documented. Polyp size was grossly estimated using open-biopsy forceps (Olympus FB-28U-1; Aomori Olympus Co., Ltd., Aomori, Japan). The polyp shape was classified as sessile (Is), semipedunculated (Isp), or pedunculated (Ip) type [12].

### Data collection

Subjects’ information, including demographics, laboratory tests, colonoscopic findings, and pathology reports, by review of electronic medical records was collected. Age was categorized into two groups: 40–44 years and 45–49 years. Body mass index (BMI) was categorized by the Western Pacific Regional Office of the World Health Organization criteria: normal (<23 kg/m^2^), overweight (23–24.9 kg/m^2^), or obese (≥25 kg/m^2^) [13]. According to the American Joint Committee’s (Joint National Committee) seventh report, blood pressure was classified as normal (<120/80 mmHg), prehypertension (120–139/80–89 mmHg), or hypertension (≥140–90 mmHg or taking antihypertensive drugs). Laboratory tests included immunoglobulin G specific for *Helicobacter pylori*, which was screened by an enzyme-linked fluorescence assay in each serum (ELFA, enzyme-linked via Vidas; bioMerieux Vitek, Inc., USA); elevated total cholesterol (≥240 mg/dL); elevated triglycerides (≥200 mg/dL); elevated low-density cholesterol (≥100 mg/dL); and low high-density lipoprotein cholesterol (HDL-c, <40 mg/dL). All lipid and lipoprotein levels were measured using a Hitachi 7600 Modular Dp-110 auto-analyzer, which included enzymatic colorimetric tests. Based on the normal reference range, serum lipid profiles were classified as normal or abnormal results.

We collected polyp data from colonoscopy reports. Advanced colorectal neoplasms were defined as follows: 1) CRC; 2) adenoma with a diameter of ≥10 mm; 3) tubular adenoma with high-grade dysplasia; and 4) tubulovillous or villous adenoma. When two or more adenoma were detected, the most advanced lesion was analyzed based on the largest diameter or advanced histology. All polyps were evaluated histologically by gastroenterology pathologists.

### Statistical analysis

Continuous variables were presented as mean ± standard deviation, and categorical variables were expressed as percentages. Continuous variables were compared using Student *t*-test, whereas categorical variables were compared using chi-square or Fisher exact tests. For prediction model development, differences between variables were compared in the training set. Before univariate analysis, continuous variables were converted to categorical variables. Variables with a *P*-value <0.1 in univariate analysis were included in subsequent multivariate regression analysis in order to select variables to be implemented in the final model. Backward elimination (i.e., removing the covariate with the largest *P* value, one at a time) was performed until we developed a final model with statistically significant covariates. We established a risk score model by excluding less significant variables in a risk assessment. We intentionally only used categorized variables that captured easy but relevant and validated information in the prediction model to develop an easy to use screening score. We used a weighted scoring system by rounding down odds ratios (ORs) to the nearest integer in the final model. For example, an OR of 1.96 was rounded to 1 and an OR of 2.26 was rounded to 2. Based on this statistical analysis, we developed a formula for predicting asymptomatic subjects aged 40–49 years with advanced neoplasm, and these subjects may be primary candidates for screening colonoscopy. To validate the model, we assessed the diagnostic accuracy of the model in the validation set. The sensitivity, specificity, positive predictive value (PPV), and negative predictive value (NPV) were calculated using the computed area under receiver operating characteristic curve (AUROC). Thereafter, we validated the diagnostic value of the scoring model. *P* < 0.05 was considered to indicate statistical significance. All analyses were performed using SPSS software (version 20.0; SPSS Inc.) and MedCalc software (version 11.1; Mariakerke) for the receiver operating characteristic analysis.

## Results

### Baseline characteristics of the total subjects

Among 2781 subjects, mean age was 44.8 ± 2.8 years, and 58.7% (1633/2781) were men. Helicobacter serology positivity was observed in 1623 (58.4%) patients. Regarding the prevalence of colorectal neoplasm, advanced neoplasm was detected in 70 (2.5%) of patients, whereas any type of colorectal neoplasm was found in 561 (20.2) of patients. Baseline characteristics of 2781 patients in the training and validation sets are summarized in Table 1. There was no statistically significant difference in patient characteristics between the training and validation sets.

**Table 1:** Clinical characteristics of subjects aged 40–49 years

Advanced neoplasm	Total subjects (*n* = 2,781)
Sex	
Male	1,633 (58.7)
Female	1,148 (41.3)
Age	44.8 ± 2.8
Age group	
40–44	1,259 (45.3)
45–49	1,522 (54.7)
Blood pressure (mmHg)	
Systolic blood pressure	120.9 ± 13.9
Diastolic blood pressure	76.3 ± 9.6
Body mass index (kg/m^2^)	23.4 ± 3.0
Anti-*H. pylori* IgG (serum)	
Positive	1,623 (58.4)
Negative	1,158 (41.6)
Total cholesterol	198.3 ± 34.1
Triglyceride	124.3 ± 81.5
LDL-cholesterol	124.0 ± 31.0
HDL-cholesterol	
Men	48.4 ± 10.4
Women	58.6 ± 13.0
Advanced neoplasm	70 (2.5)
Overall neoplasm	561 (20.2)

*LDL* low-density lipoprotein, *HDL* high-density lipoprotein, *H. pylori, Helicobacter pylori*

### Clinicopathological characteristic of the colorectal neoplasm

Details of the clinicopathological findings of neoplasm are summarized in Table 2. Among 561 neoplasms, 64 (11.4%) were larger than 10 mm, 537 (95.7%) had a low-grade tubular adenoma, and 24 (4.3%) had advanced neoplasm, including 2 patients (0.4%) with cancer.

**Table 2:** Overall colorectal neoplasm in subjects aged 40–49 years

	*N* = 561	%
Number		
One	312	55.7
Two	152	27.1
Three or more	96	17.2
Size (mm)^*^		
< 10	496	88.6
≥ 10	64	11.4
Histology^*^		
Low-grade tubular adenoma	537	95.7
High-grade tubular, villous adenoma	22	3.9
Adenocarcinoma	2	0.4
Shape^*^		
Sessile	384	66.9
Semipedunculated	158	28.3
Pedunculated	27	4.8
Location^*^		
Ascending colon	124	22.3
Transverse colon	131	23.4
Descending colon	80	14.3
Rectosigmoid	225	40.0

^*^Results are summarized according to the most advanced lesion

### Factors associated with advanced colorectal neoplasm in the development dataset

Univariate analysis showed that male sex (*P* ≤ 0.001), the obese group (*P* = 0.016), positive serology of *H. pylori* (*P* = 0.009), low HDL level (*P* ≤ 0.001), and high triglyceride level (*P* ≤ 0.001) were significantly associated with the presence of advanced neoplasm. Moreover, the older age group (45–49 years) was marginally associated with the presence of advanced neoplasm (*P* = 0.053). Details of the statistical analysis are summarized in Table 3. In subsequent multivariate analysis, the older age group (OR 1.967, 95% CI 1.191–3.749, *P* = 0.040), male sex (OR 2.763, 95% CI 1.032–6.409, *P* = 0.018), positive serology of *H. pylori* (OR 2.262, 95% CI 1.108–4.621, *P* = 0.025), low HDL level (OR 2.219, 95% CI 1.073–4.308, *P* = 0.031), and high triglyceride level (OR 1.967, 95% CI 1.143–4.252, *P* = 0.018) were identified as independent factors for advanced neoplasm. BMI (body mass index) was not significant in multivariable analysis. Details of these values are depicted in Table 4.

**Table 3:** Comparison of variables based on the presence of advanced neoplasm in the training set

Advanced neoplasm	Development set (*n* = 1,844)		*P*-value
	No (*n* = 1,798)	Yes (*n* = 46)	
Sex			<0.001
Male	1,044 (96.4)	39 (3.6)	
Female	754 (99.1)	7 (0.9)	
Age group			0.053
40–44	805 (98.3)	14 (1.7)	
45–49	993 (96.9)	32 (3.1)	
Blood pressure (mmHg)			0.151
Normal	809 (98.2)	15 (1.8)	
Pre-HTN	741 (97.2)	21 (2.8)	
HTN	248 (96.1)	10 (3.9)	
BMI (kg/m^2^)			0.016
< 23.0	845 (98.6)	12 (1.4)	
< 25.0	458 (96.8)	15 (3.2)	
25.0	495 (96.3)	19 (3.7)	
Anti-*H. pylori* IgG			0.009
Positive	1,062 (96.7)	36 (3.3)	
Negative	736 (98.7)	10 (1.3)	
Total cholesterol			0.755
Normal	1,529 (97.8)	40 (2.2)	
Elevation (≥240 mg/dL)	222 (97.5)	5 (2.5)	
LDL-c			0.237
Normal	140 (100)	0	
Elevation (≥100 mg/dL)	998 (99.0)	10 (1.0)	
HDL-c (<40 mg/dL)			<0.001
Normal	1,554 (98.1)	30 (1.9)	
Low (<40 mg/dL)	244 (93.8)	16 (6.2)	
Triglyceride			<0.001
Normal	1,588 (98.0)	32 (2.0)	
Elevation (≥200 mg/dL)	210 (93.8)	14 (6.3)	

*HTN* hypertension, *BMI* body mass index, *LDL-c* low-density lipoprotein, *HDL-c* high-density lipoprotein, *H. pylori, Helicobacter pylori*

**Table 4:** Multivariate regression analysis in the development set

	Odds ratio (95% CI)	*P*-value	Score assigned
Older age (45–49 years)	1.967 (1.191–0.040)	0.040	1
Male sex	2.763 (1.032–0.018)	0.018	2
Positive serology of *H. pylori*	2.262 (1.108–0.025)	0.025	2
High triglyceride level	2.219 (1.073–0.031)	0.031	2
Low HDL-c	2.136 (1.143–4.252)	0.018	2

*CI* confidence interval, *H. pylori, Helicobacter pylori*, *HDL-c* high-density lipoprotein cholesterol

A simple scoring model for advanced colorectal neoplasm = Age [0: 40–44, 1: 45–49 years] × 1 + Sex [0: female, 1: male] × 2 + Serology of *H. pylori* [0: negative, 1: positive] × 2 + Triglyceride level [0: normal range, 1: high] × 2 + High-density lipoprotein level [0: normal range, 1: low] × 2

### Development of a simple scoring model for predicting advanced neoplasm

A simple scoring model was constructed based on five independently significant variables in multivariate analysis by using rounded down ORs of each variable (Table 4). The final model is as follows:

***A simple scoring model for advanced colorectal neoplasm*** = Age [0: 40–44, 1: 45–49 years] × 1 + Sex [0: female, 1: male] × 2 + Serology of *H. pylori* [0: negative, 1: positive] × 2 + High triglyceride level [0: normal range, 1: high] × 2 + Low HDL level [0: normal range, 1: low] × 2

**Fig. 1:**
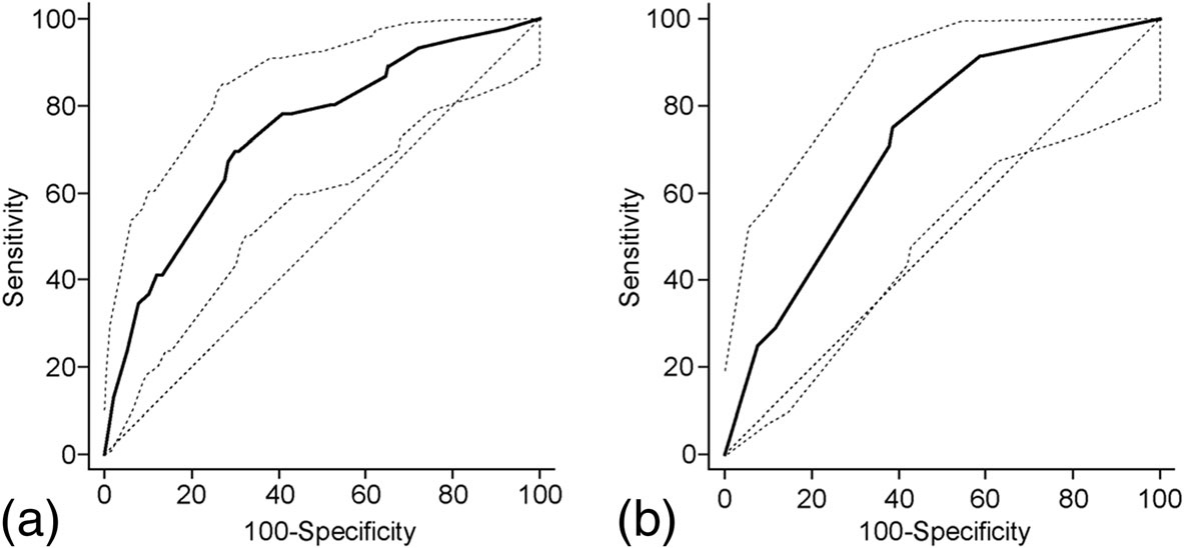
Area under the receiver operating characteristic (AUROC) curve of the simple scoring model for advanced neoplasm in subjects aged 40–49 years. An AUROC of 0.74 (95% confidence interval [CI] 0.717–0.757) in development set (**a**) and an AUROC of 0.72 (95% CI 0.0695–0.753) in the validation set (**b**)

The range of the total score for this risk model was 0–9. This model yielded an AUROC of 0.74 for predicting advanced neoplasm in the development set (Fig. 1a).

### Validation of the scoring model in the validation set

**Fig. 2:**
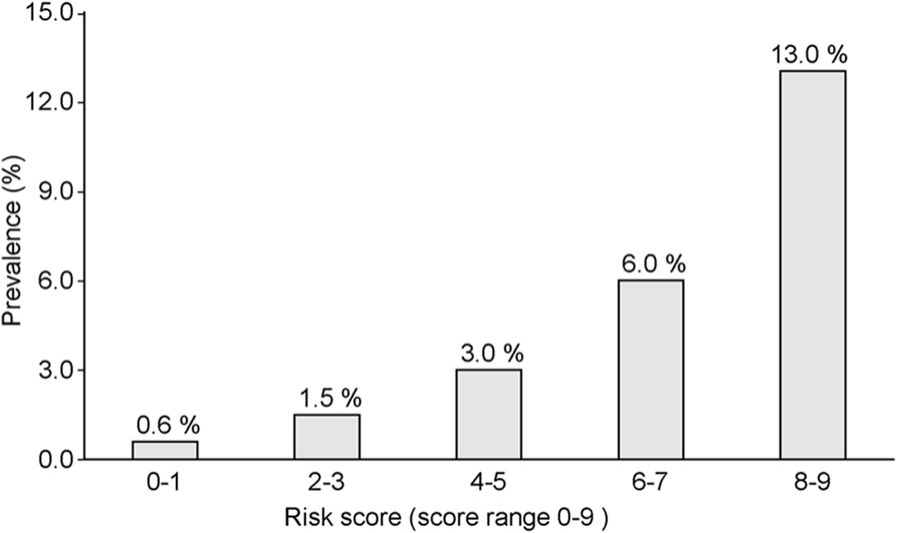
Estimated prevalence of advanced neoplasm according to the risk score in the validation set. The proportion of subjects with scores of 0–1, 2–3, 4–5, 6–7, and 8–9 correspond to 0.6, 1.5, 3.0, 6.0, and 13.0%, respectively. A cutoff point of ≥4 (sensitivity 79%, specificity 58%) indicates that 43% of subjects are at high risk for advanced colorectal neoplasm

We investigated the predicted value of different total score cut points in the validation sets (Table 5). A cutoff point of 4 was selected because it results in the highest value for the AUROC to indicate an individual at high risk for advanced neoplasm. In the validation set, this cutoff point designated 43.1% of subjects at high risk for advanced colorectal neoplasm, and yielded a sensitivity of 79.2%, specificity of 57.8%, PPV of 4.7%, and NPV of 99.1% with an AUROC of 0.72 (Fig. 1b). The prevalence of advanced neoplasm gradually increased as the total risk score increased, and these findings are depicted in Fig. 2.

**Table 5:** Performance of the simple scoring model in the validation datasets (*n* = 937)

Score	High risk (%)	Sensitivity	Specificity	PPV	NPV	AUROC
≥0	100	100	0	2.8	0	0.50
≥1	90.2	100	10.1	2.8	100	0.55
≥2	81.1	95.8	19.3	3.0	99.4	0.58
≥3	64.2	87.5	36.4	3.5	99.1	0.62
≥4	43.1	79.2	57.8	4.7	99.1	0.72
≥5	30.3	62.5	70.5	5.3	98.6	0.68
≥6	13.6	37.5	87.1	7.1	98.1	0.62
≥7	8.0	25.0	92.4	8.0	97.9	0.59
≥8	3.4	8.3	96.7	6.2	97.6	0.53
≥9	1.5	4.2	98.6	7.1	97.5	0.51

*PPV* positive predictive value, *NPV* negative predictive value, *AUROC* area under the receiver operating characteristic curve

## Discussion

To the best of our knowledge, our study is the first study to propose a prediction model for advanced neoplasm in the 40–49-year-old population. In the 40–49-year-old group, an increased risk of advanced neoplasm was associated with those aged ≥45 years, male sex, positive serology of *H. pylori*, and high triglyceride and low HDL levels. We developed a simple scoring model for predicting advanced colorectal neoplasm (range 0–9), and a cutoff point of ≥4 in this model yielded a sensitivity of 78% and specificity of 56% (AUROC = 0.74) (data not shown). Comparable results were obtained in the validation datasets (sensitivity 79%, specificity 58%, and AUROC = 0.72).

It has been reported that the prevalence of advanced colorectal neoplasm in individuals aged 40–49 years is 1.3–3.5% [10, 14–17]. Similar to these results, our study showed a 2.5% prevalence for advanced neoplasm in an asymptomatic population. Currently, advanced adenoma is regarded as the most feasible target for CRC screening [2, 6, 7]. Based on our prediction model, the prevalence of advanced neoplasm gradually increased as the risk score increased. This finding suggests that our prediction model can be a reliable tool for identifying target subjects for CRC screening among persons aged 40–49 years. Our prediction model produced moderate accuracy for predicting advanced neoplasm, producing an AUROC value of 0.72 in the validation cohort.

Regarding age-related risk stratification, our study showed that the risk for advanced neoplasm was approximately two times higher in the 45–49 age group than in the 40–44 age group. This finding suggests that further subgroup classification can be helpful in identifying the population at risk within the 40–49 age group, and it was eventually incorporated into our prediction model. Similar to our results, Hong et al. [10] also reported that the 45–49 age group had a high adjusted OR of 1.68 compared to the 40–44 age group for detecting advanced neoplasm.

Among lipid profile indices, we found that hypertriglyceridemia and low HLD-c were independently associated with advanced neoplasm. In accordance with our findings, a previous study showed that higher levels of serum triglyceride were significantly associated with an increasing prevalence of both non-advanced and advanced colorectal adenoma in a population aged 40–79 years [18]. Concerning the association between hypertriglyceridemia and advanced neoplasm, although the exact mechanism is currently unclear, several mechanisms have been suggested. First, hyperinsulinemia and insulin resistance is affected by apoptosis, thereby a lower rate of apoptosis in normal colonic mucosa is associated with the carcinogenesis process, and they may develop into colorectal adenoma, or even cancer [19–21]. Second, hypertriglyceridemia can also increase proinflammatory cytokine levels, and damage deoxyribonucleic acid. These also affect carcinogenesis through abnormal growth, apoptosis, and the proliferation of colorectal cells [22–24].

Meanwhile, similar to our results of association between a low HDL-c level and the risk of advanced neoplasm, trends for an increased prevalence of advanced adenoma with decreasing levels of HDL cholesterol was also found in previously reported data [18]. Other studies have also shown that a reduction in HDL cholesterol levels slightly increases the risk of adenomatous colon polyps, and consequently colon cancer [25, 26]. Although the underlying mechanism for the relationship between a low HLD level and the risk of advanced neoplasm is still elusive, a low HDL level may result from interactions with triglyceride and HDL cholesterol, and the aforementioned possible molecular mechanism associated with hypertriglyceridemia and colonic adenoma may also be involved in these processes. Further extensive molecular biological studies are needed to clarify the association between dyslipidemia and the risk of colonic neoplasm.

An association between colorectal neoplasm and *H. pylori* infection has been steadily suggested, and meta-analysis data also demonstrated that *H. pylori* infection increase the risk of colorectal neoplasm by 1.4–1.6 times [27]. In addition, a recent large cross-sectional study showed that the OR of the group with *H. pylori* gastritis is about 1.24–2.35 [28]. In accordance with these observations, our study also showed that patients with *H. pylori* infection are 2.26 times more likely to have advanced neoplasm compared to patients without *H. pylori* infection in subjects aged 40–49 years. Although the relationship between *H. pylori* infection and increased risk of colorectal adenoma is unclear, several pathogenic mechanisms have been suggested. Increased gastrin induced by *H. pylori* infection may contribute to carcinogenesis by cell proliferation in the colon mucosa, and *H. pylori* itself can act on the colorectal epithelium through inflammatory responses and affect polyp growth or promote mucosal dysplasia [29–31].

There are several advantages to our study. First, our prediction model was developed based on a large study population that included 1844 and 937 asymptomatic subjects in the training and validation cohort, respectively. Second, our model consisted of basic demographic factors and readily available serologic indices. Moreover, the final score of our model can be calculated in daily practice; thus, it has potential for high clinical utility in selective screening colonoscopy among the 40–49-year-old population. Lastly, our model allows for the adjustment of a cutoff value for subgroup targeting based on population characteristics and endoscopic resources.

Several limitations of our study should be mentioned. First, this study was performed in a health promotion center of a single university hospital in Korea. Therefore, generalizability of our results to the general population and other ethnicities is uncertain. Second, our study did not include lifestyle factors such as drinking, smoking, physical activity, and medication history. It has been reported that these lifestyle factors and some drugs are associated with the development of colorectal neoplasm [32–34]. Nevertheless, previous data have shown that some lifestyle factors, including smoking, alcohol, and medication, use were not significant predictors of advanced neoplasm in average risk screenees aged 40–49 years [10]. Hypertriglyceridemia is associated with alcohol consumption and a degree of smoking. Moreover, low HDL levels are related with a smoking habit, and its value in serum can be increased by regular exercise [35–38]. These observations imply that lipid indices can partly reflect lifestyle characteristics of each single person, and these lipid indices are incorporated in our prediction model. Finally, the retrospective nature of this study poses some limitations. However, our data were gathered prospectively, and our model largely consisted of objective results of serology, which were measured on the same day of colonoscopy. Thus, the influence of this limitation seems to be minimal. Further prospective studies incorporating diverse demographic, clinical, and serological parameters should be conducted.

## Conclusions

In conclusion, older age (45–49 years), male sex, positive serology of *H. pylori*, and high triglyceride and low HDL levels were identified as independent risk factors for advanced colorectal neoplasm. A simple scoring model that consists of five parameters may be useful for selecting patients who benefit from screening colonoscopy in asymptomatic persons aged 40–49 year. We hope that researchers will evaluate the performance of our prediction model in an independent population to confirm and validate our results.
